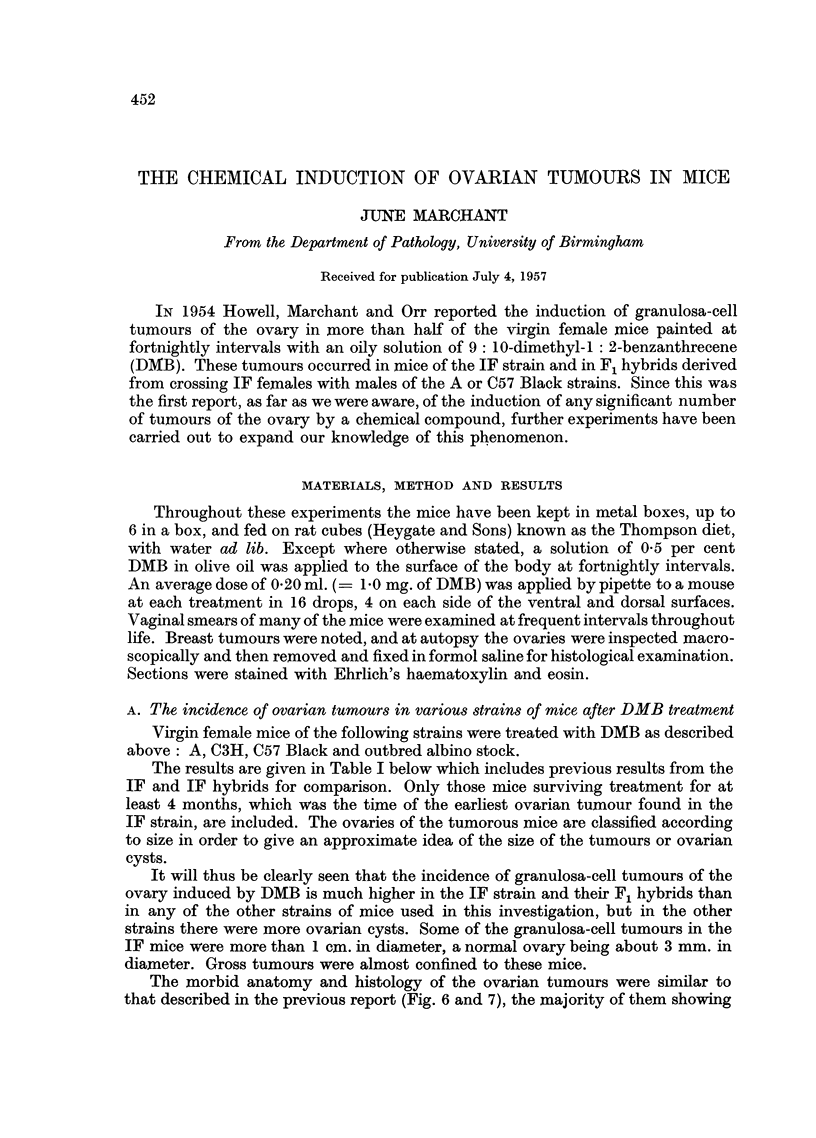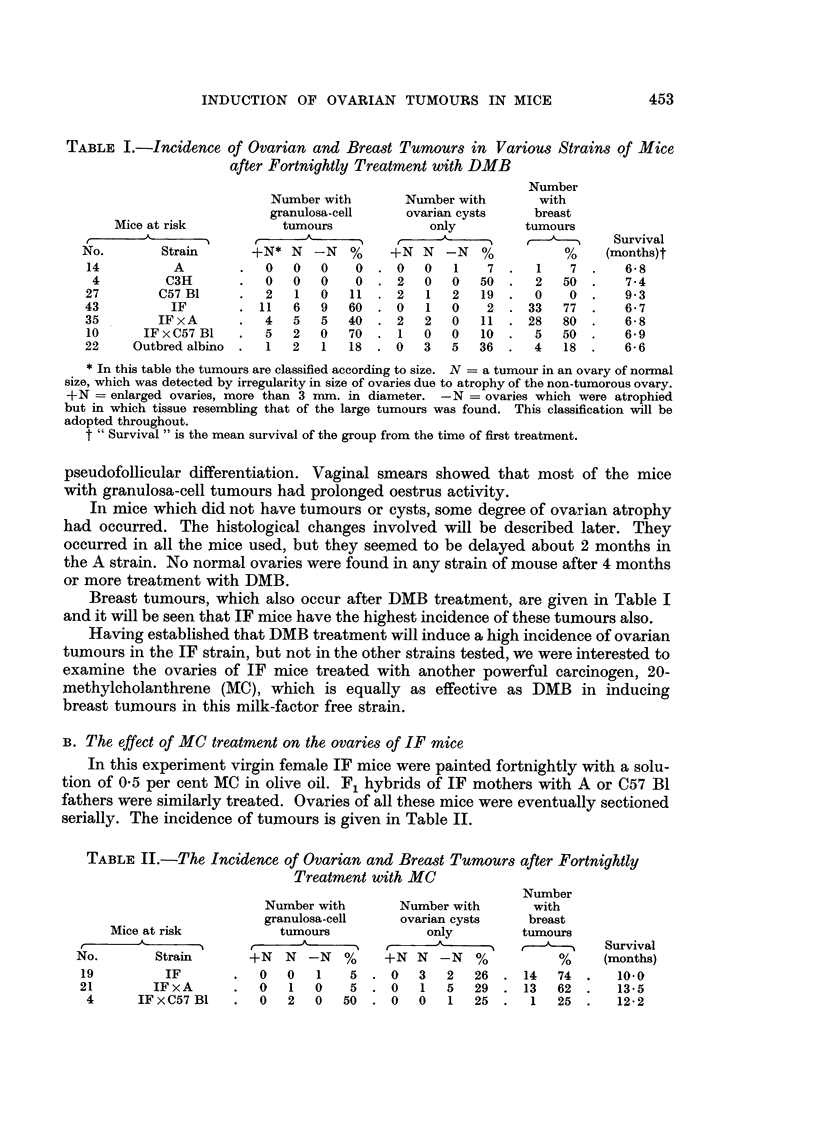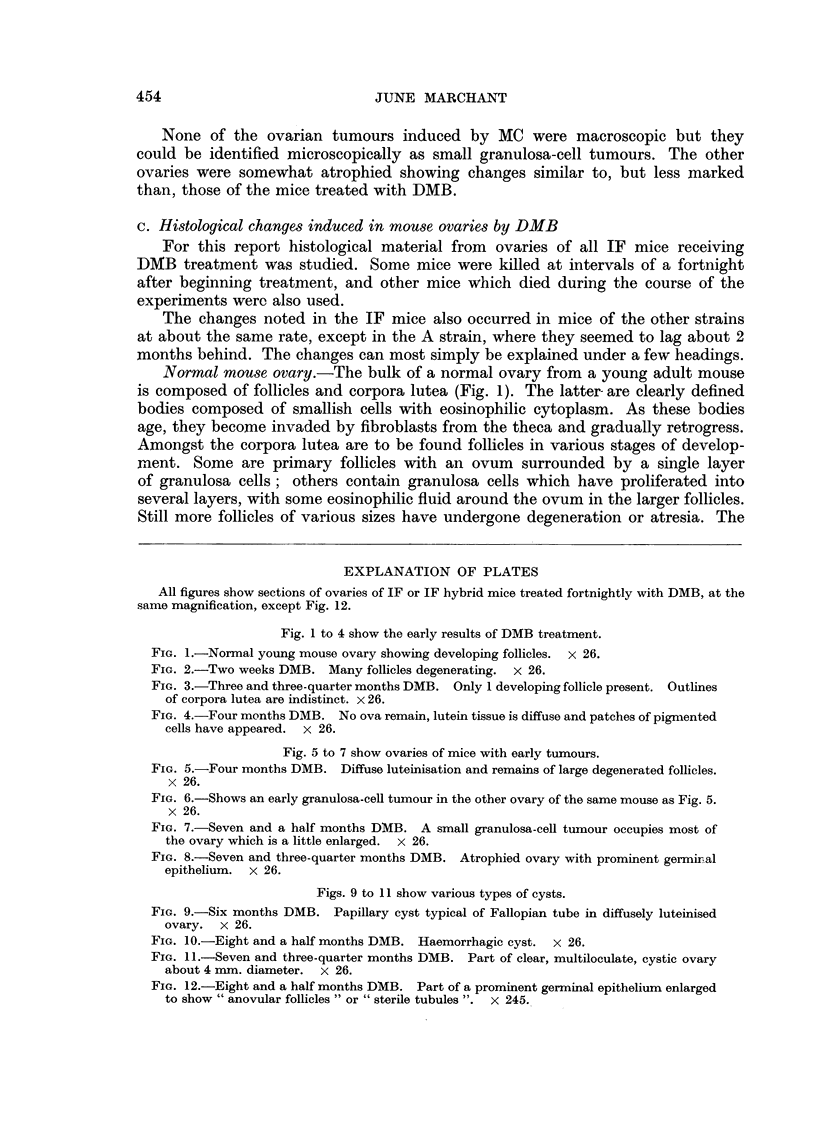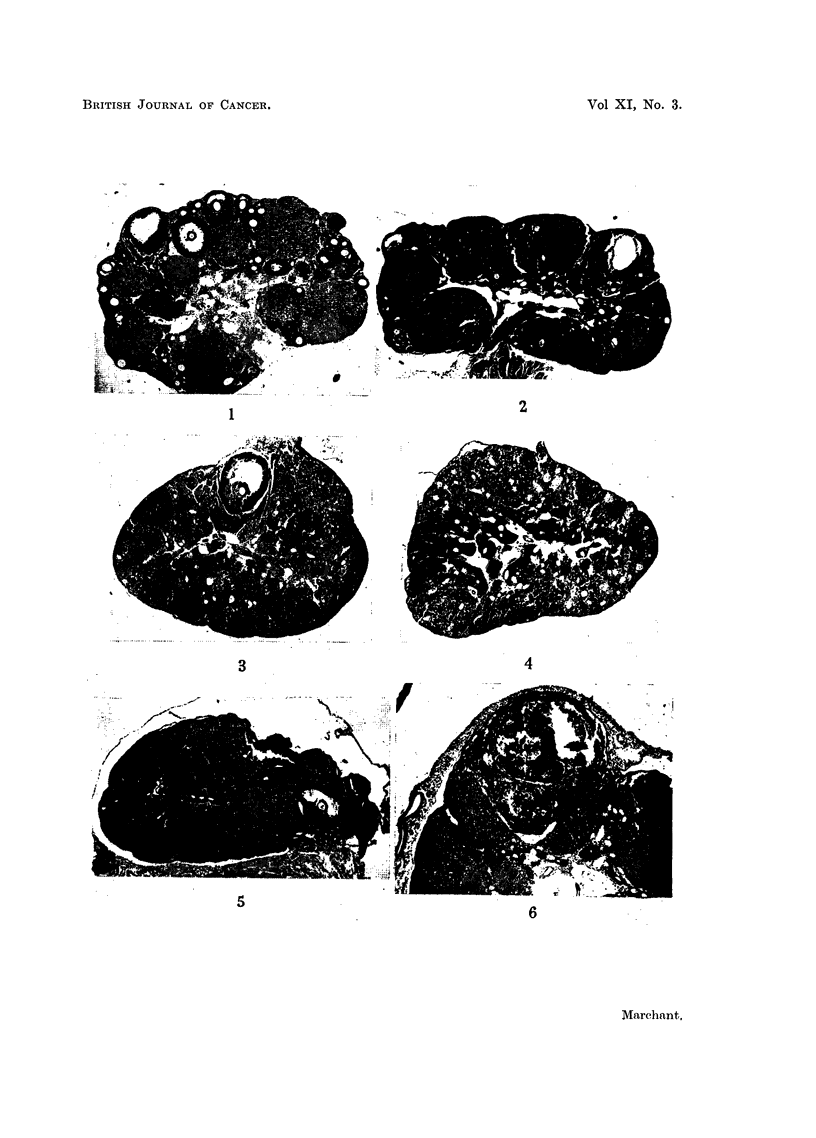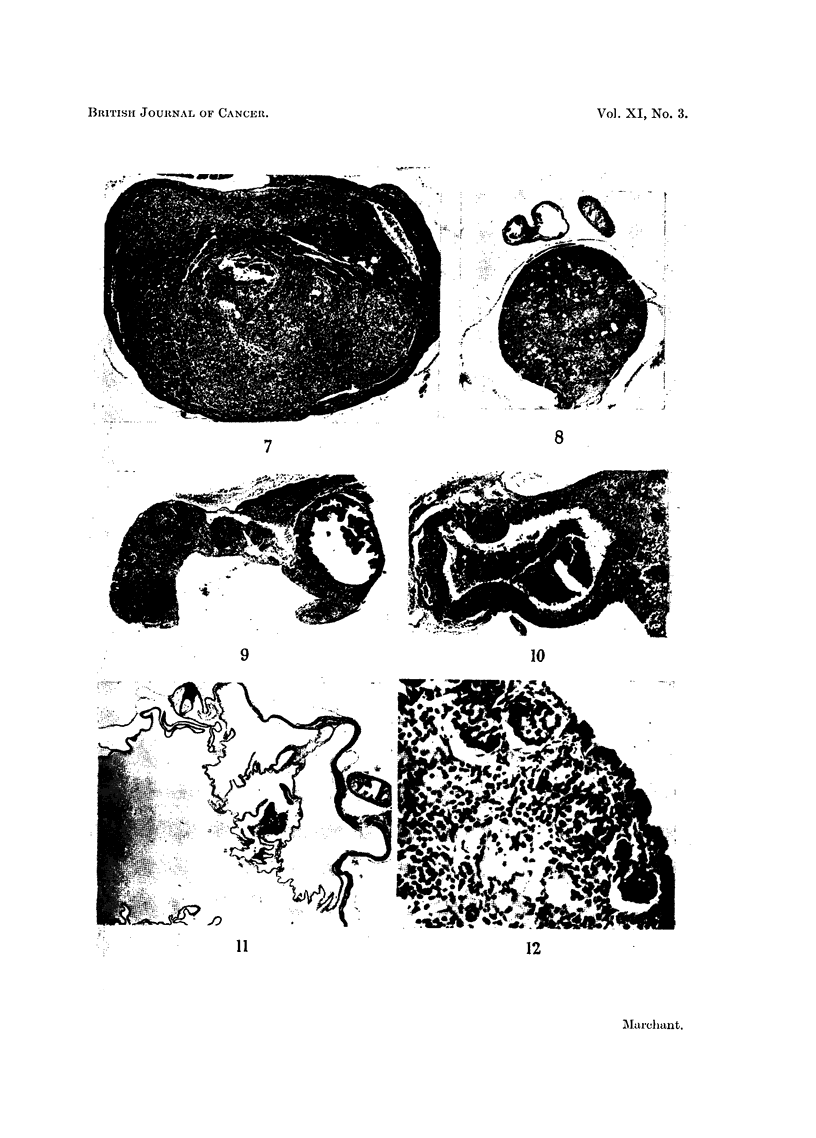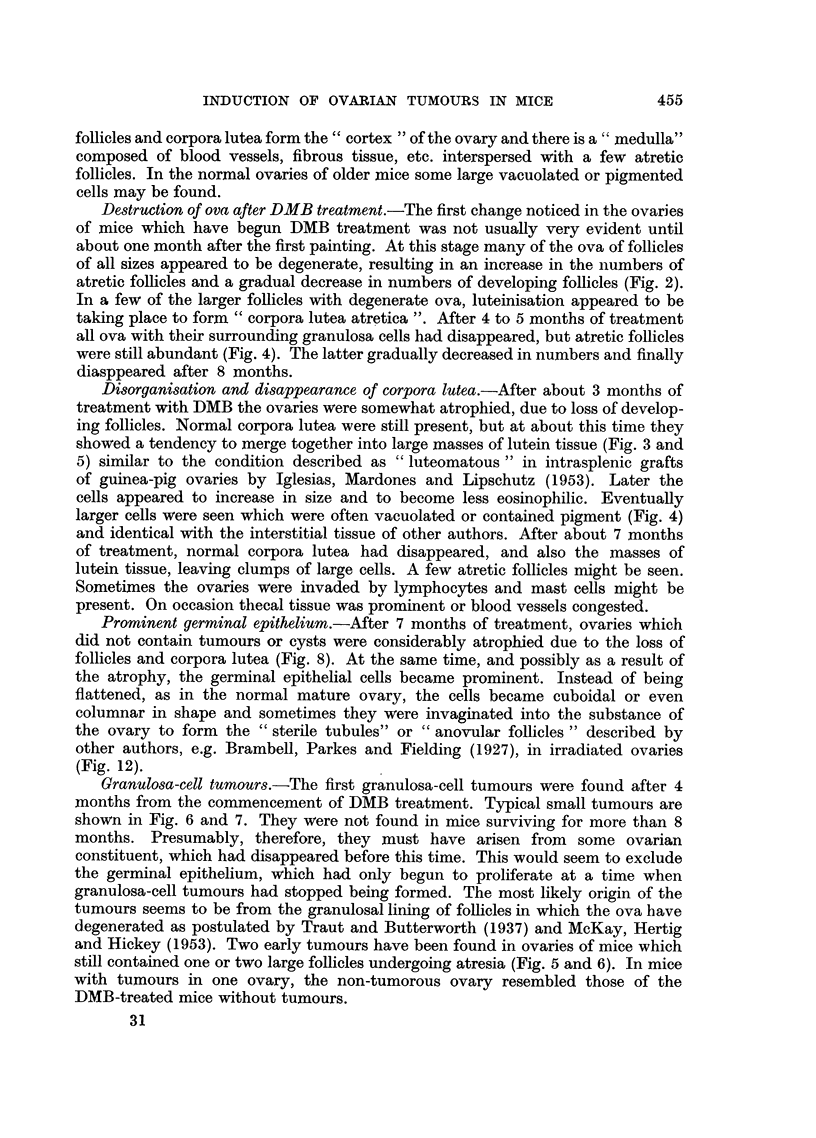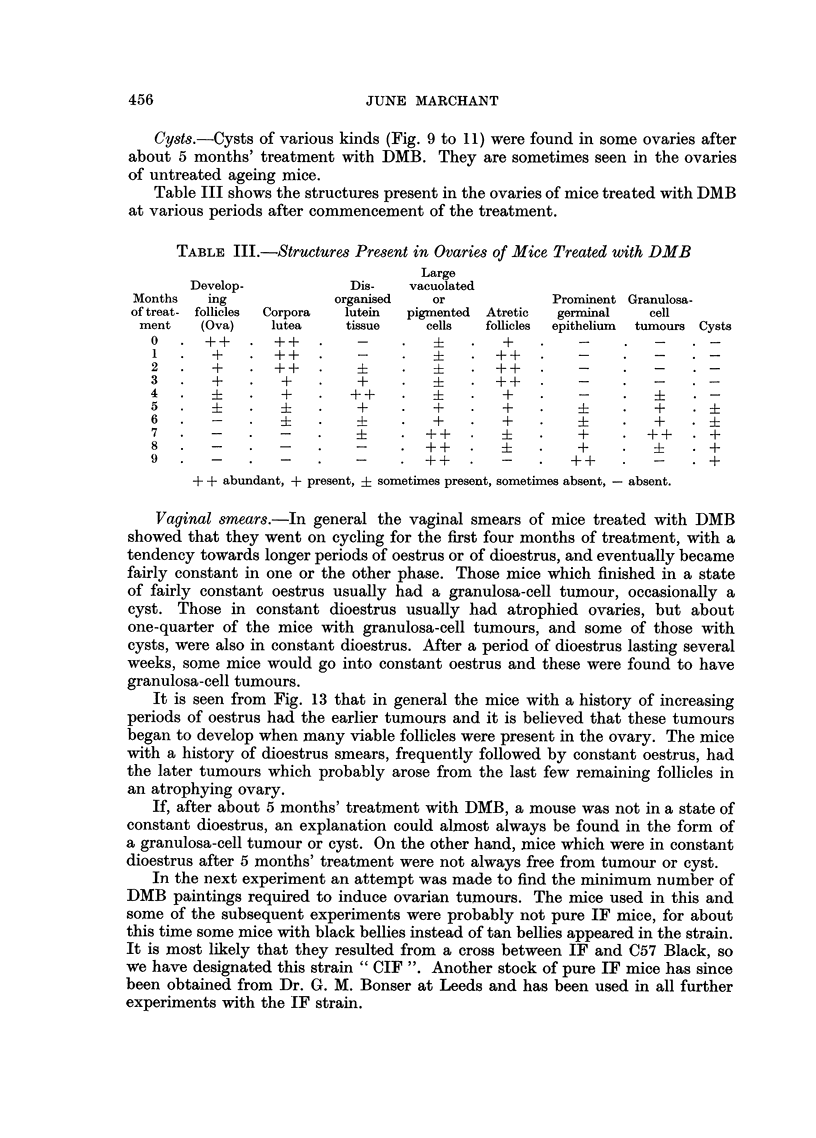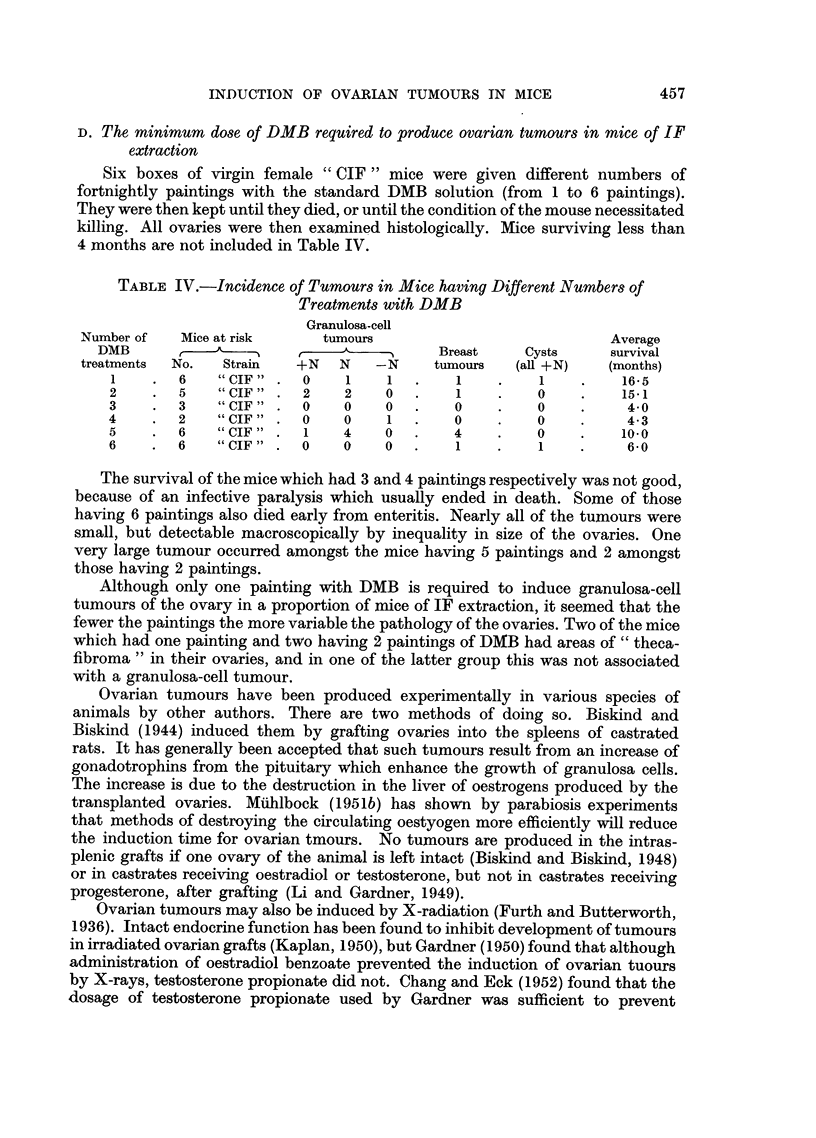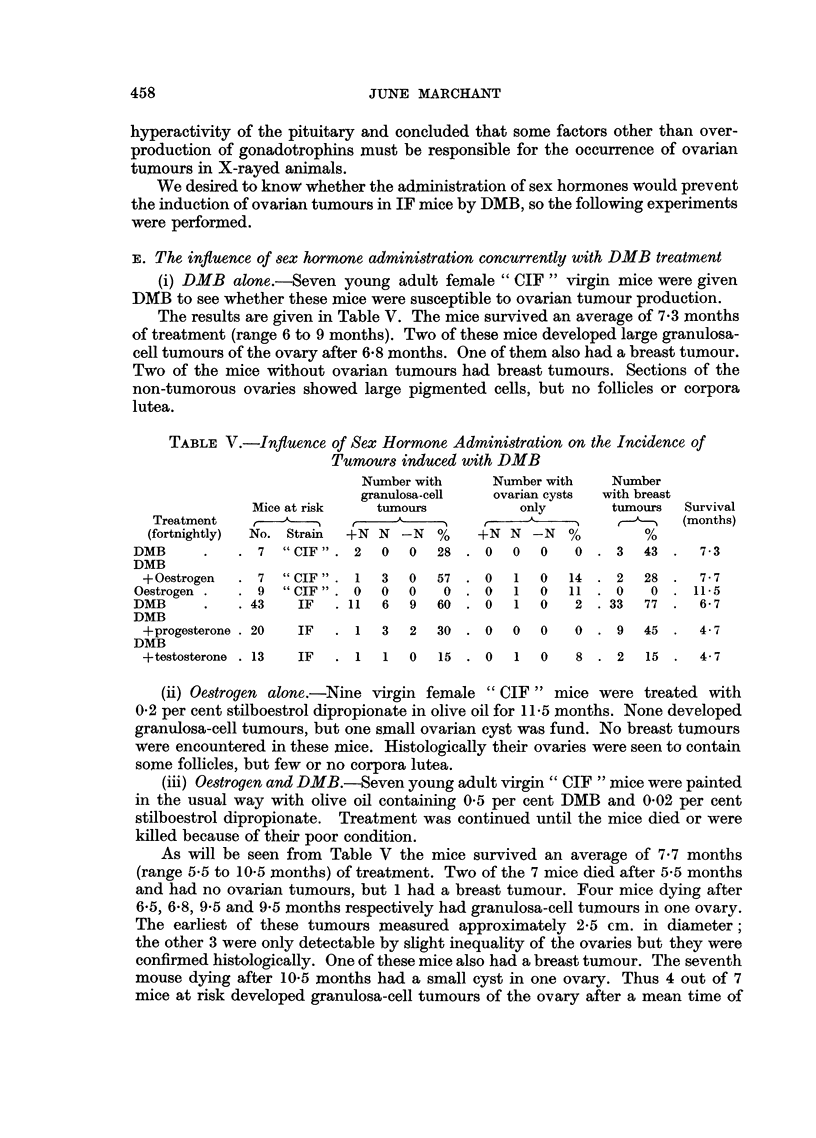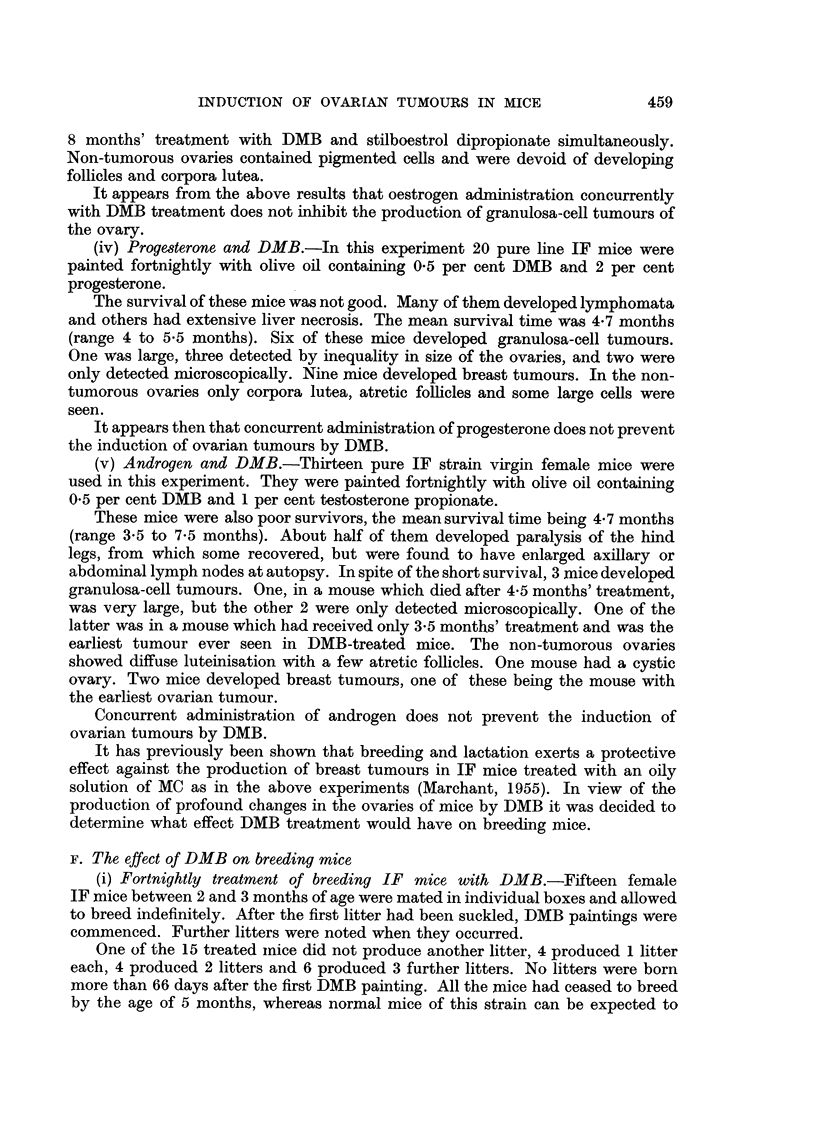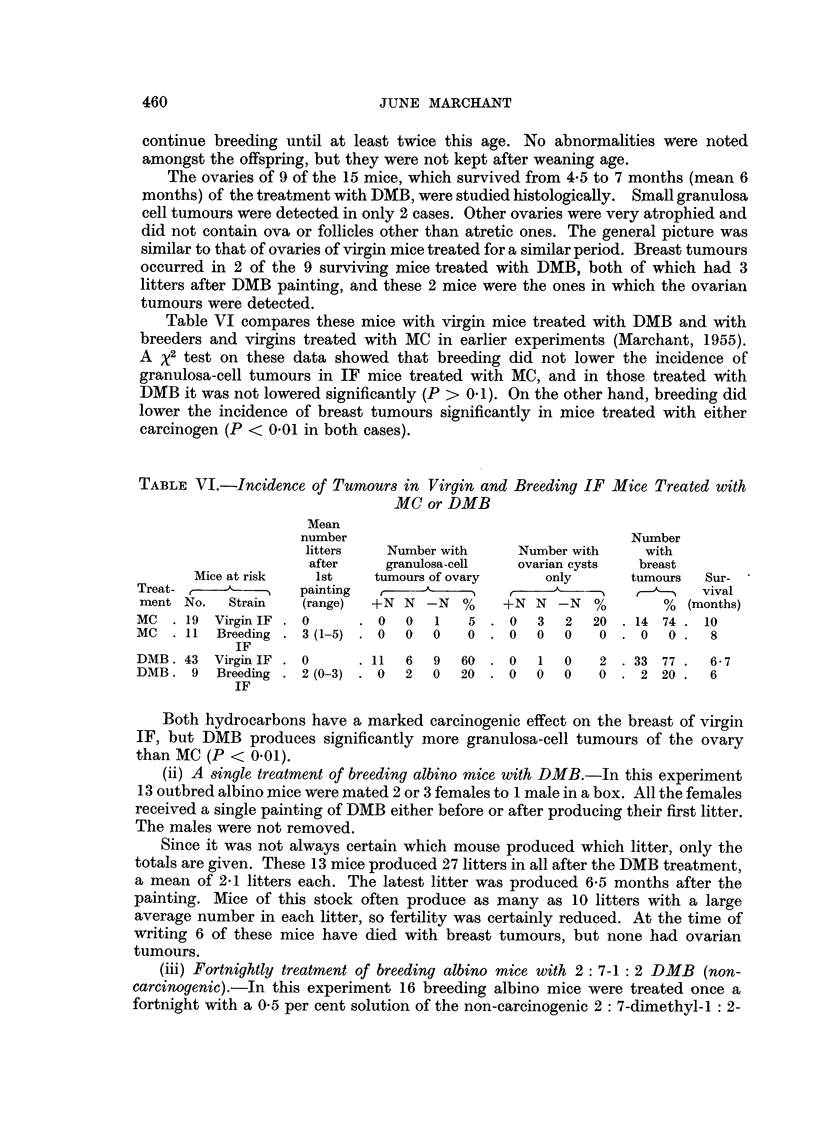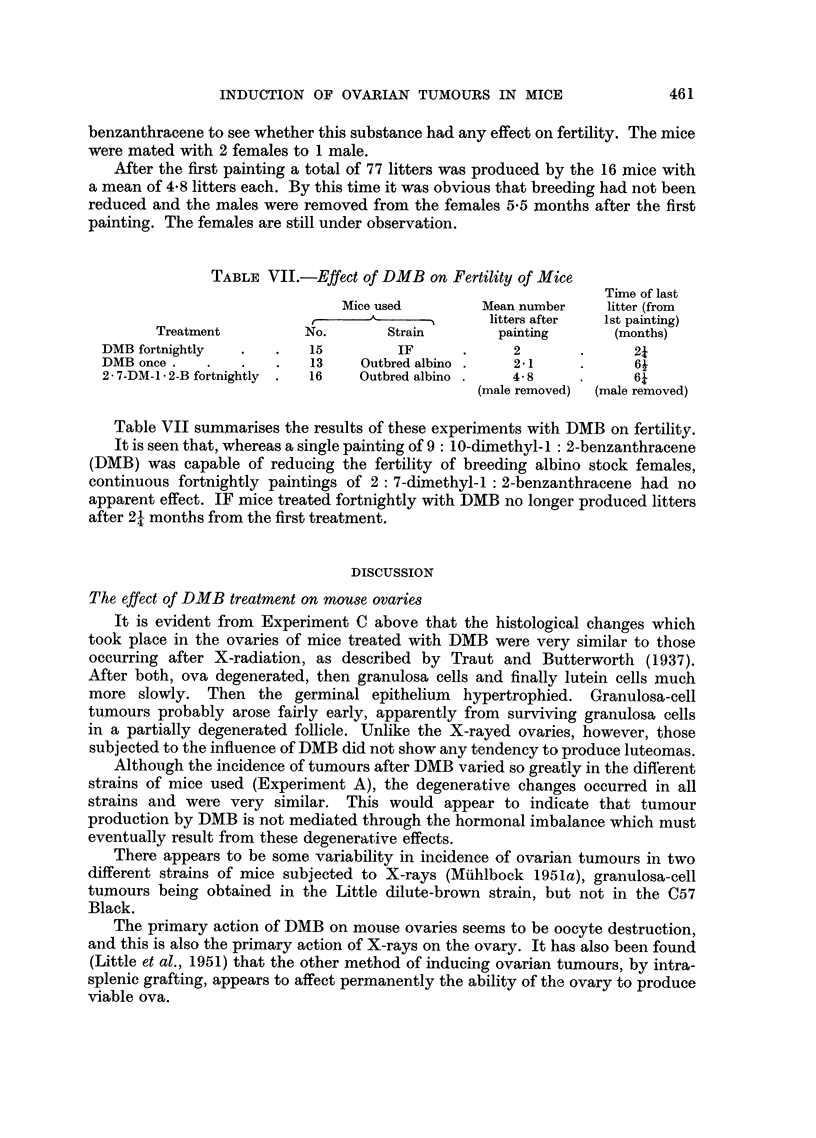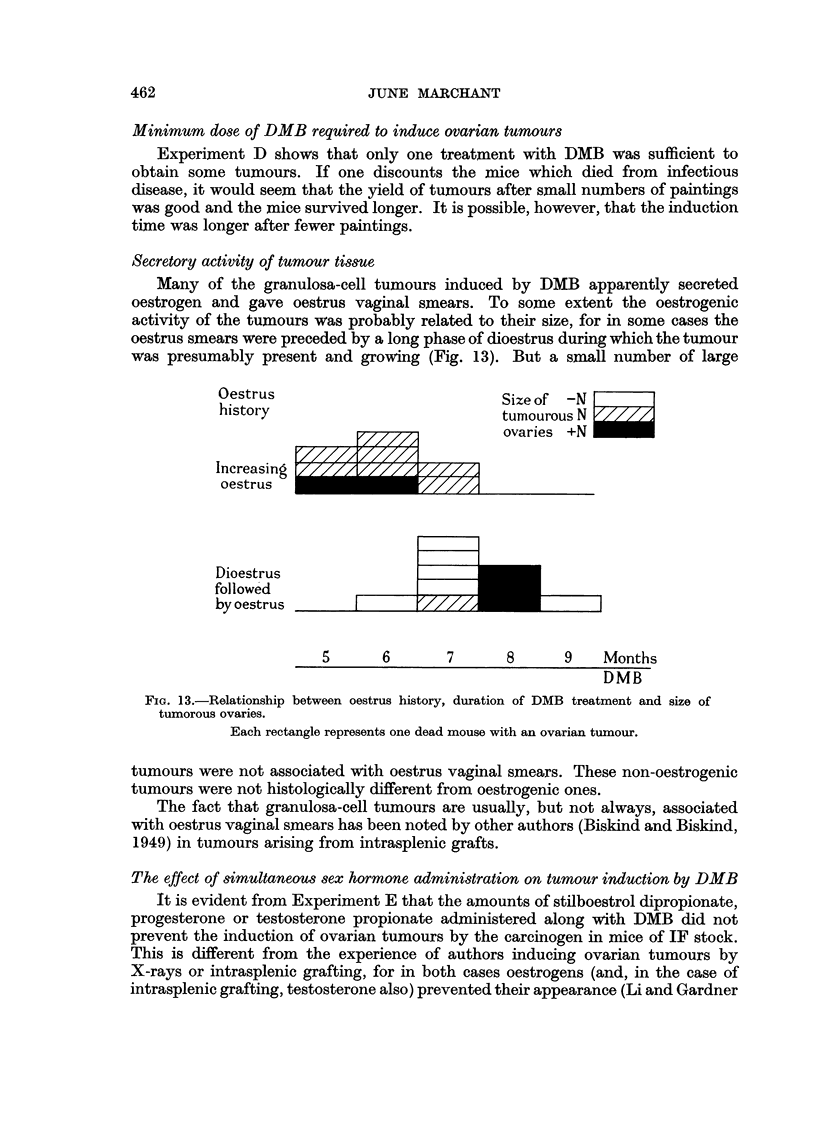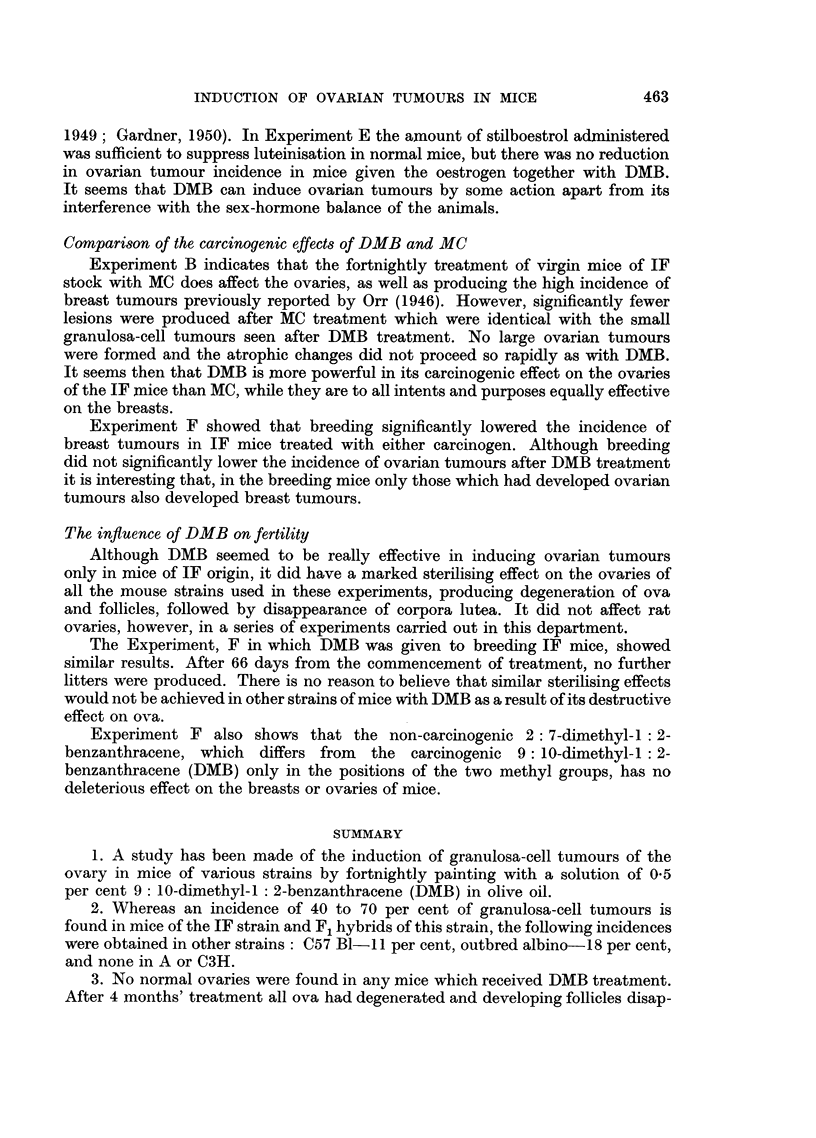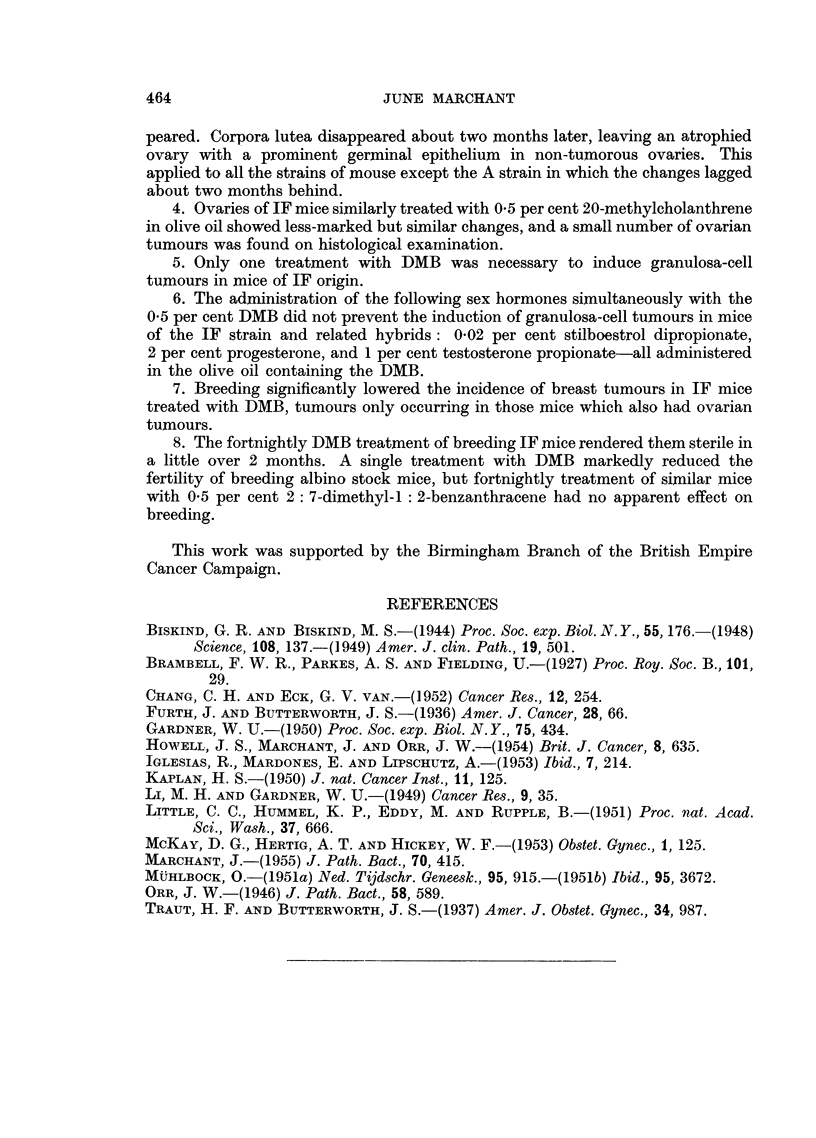# The Chemical Induction of Ovarian Tumours in Mice

**DOI:** 10.1038/bjc.1957.53

**Published:** 1957-09

**Authors:** June Marchant

## Abstract

**Images:**


### THE CHEMICAL INDUCTION OF OVARIAN TUMOURS IN MICE

JUNE MARCHANT

*From the Department of Pathology, University of Birmingham*

Received for publication July 4, 1957

In 1954 Howell, Marchant and Orr reported the induction of granulosa-cell tumours of the ovary in more than half of the virgin female mice painted at fortnightly intervals with an oily solution of 9 : 10-dimethyl-1 : 2-benzanthrecene (DMB). These tumours occurred in mice of the IF strain and in $F_1$ hybrids derived from crossing IF females with males of the A or C57 Black strains. Since this was the first report, as far as we were aware, of the induction of any significant number of tumours of the ovary by a chemical compound, further experiments have been carried out to expand our knowledge of this phenomenon.

### MATERIALS, METHOD AND RESULTS

Throughout these experiments the mice have been kept in metal boxes, up to 6 in a box, and fed on rat cubes (Heygate and Sons) known as the Thompson diet, with water *ad lib.* Except where otherwise stated, a solution of 0·5 per cent DMB in olive oil was applied to the surface of the body at fortnightly intervals. An average dose of 0·20 ml. (= 1·0 mg. of DMB) was applied by pipette to a mouse at each treatment in 16 drops, 4 on each side of the ventral and dorsal surfaces. Vaginal smears of many of the mice were examined at frequent intervals throughout life. Breast tumours were noted, and at autopsy the ovaries were inspected macroscopically and then removed and fixed in formol saline for histological examination. Sections were stained with Ehrlich's haematoxylin and eosin.

### A. *The incidence of ovarian tumours in various strains of mice after DMB treatment*

Virgin female mice of the following strains were treated with DMB as described above : A, C3H, C57 Black and outbred albino stock.

The results are given in Table I below which includes previous results from the IF and IF hybrids for comparison. Only those mice surviving treatment for at least 4 months, which was the time of the earliest ovarian tumour found in the IF strain, are included. The ovaries of the tumorous mice are classified according to size in order to give an approximate idea of the size of the tumours or ovarian cysts.

It will thus be clearly seen that the incidence of granulosa-cell tumours of the ovary induced by DMB is much higher in the IF strain and their $F_1$ hybrids than in any of the other strains of mice used in this investigation, but in the other strains there were more ovarian cysts. Some of the granulosa-cell tumours in the IF mice were more than 1 cm. in diameter, a normal ovary being about 3 mm. in diameter. Gross tumours were almost confined to these mice.

The morbid anatomy and histology of the ovarian tumours were similar to that described in the previous report (Fig. 6 and 7), the majority of them showing

TABLE I.—*Incidence of Ovarian and Breast Tumours in Various Strains of Mice after Fortnightly Treatment with DMB*

| Mice at risk | | Number with granulosa-cell tumours | | | | Number with ovarian cysts only | | | | Number with breast tumours | | Survival (months)† |
|---|---|---|---|---|---|---|---|---|---|---|---|---|
| No. | Strain | +N* | N | −N | % | +N | N | −N | % | | % | |
| 14 | A | 0 | 0 | 0 | 0 | 0 | 0 | 1 | 7 | 1 | 7 | 6·8 |
| 4 | C3H | 0 | 0 | 0 | 0 | 2 | 0 | 0 | 50 | 2 | 50 | 7·4 |
| 27 | C57 Bl | 2 | 1 | 0 | 11 | 2 | 1 | 2 | 19 | 0 | 0 | 9·3 |
| 43 | IF | 11 | 6 | 9 | 60 | 0 | 1 | 0 | 2 | 33 | 77 | 6·7 |
| 35 | IF×A | 4 | 5 | 5 | 40 | 2 | 2 | 0 | 11 | 28 | 80 | 6·8 |
| 10 | IF×C57 Bl | 5 | 2 | 0 | 70 | 1 | 0 | 0 | 10 | 5 | 50 | 6·9 |
| 22 | Outbred albino | 1 | 2 | 1 | 18 | 0 | 3 | 5 | 36 | 4 | 18 | 6·6 |

* In this table the tumours are classified according to size. *N* = a tumour in an ovary of normal size, which was detected by irregularity in size of ovaries due to atrophy of the non-tumorous ovary. +N = enlarged ovaries, more than 3 mm. in diameter. −N = ovaries which were atrophied but in which tissue resembling that of the large tumours was found. This classification will be adopted throughout.

† " Survival " is the mean survival of the group from the time of first treatment.

pseudofollicular differentiation. Vaginal smears showed that most of the mice with granulosa-cell tumours had prolonged oestrus activity.

In mice which did not have tumours or cysts, some degree of ovarian atrophy had occurred. The histological changes involved will be described later. They occurred in all the mice used, but they seemed to be delayed about 2 months in the A strain. No normal ovaries were found in any strain of mouse after 4 months or more treatment with DMB.

Breast tumours, which also occur after DMB treatment, are given in Table I and it will be seen that IF mice have the highest incidence of these tumours also.

Having established that DMB treatment will induce a high incidence of ovarian tumours in the IF strain, but not in the other strains tested, we were interested to examine the ovaries of IF mice treated with another powerful carcinogen, 20-methylcholanthrene (MC), which is equally as effective as DMB in inducing breast tumours in this milk-factor free strain.

B. *The effect of MC treatment on the ovaries of IF mice*

In this experiment virgin female IF mice were painted fortnightly with a solution of 0·5 per cent MC in olive oil. $F_1$ hybrids of IF mothers with A or C57 Bl fathers were similarly treated. Ovaries of all these mice were eventually sectioned serially. The incidence of tumours is given in Table II.

TABLE II.—*The Incidence of Ovarian and Breast Tumours after Fortnightly Treatment with MC*

| Mice at risk | | Number with granulosa-cell tumours | | | | Number with ovarian cysts only | | | | Number with breast tumours | | Survival (months) |
|---|---|---|---|---|---|---|---|---|---|---|---|---|
| No. | Strain | +N | N | −N | % | +N | N | −N | % | | % | |
| 19 | IF | 0 | 0 | 1 | 5 | 0 | 3 | 2 | 26 | 14 | 74 | 10·0 |
| 21 | IF×A | 0 | 1 | 0 | 5 | 0 | 1 | 5 | 29 | 13 | 62 | 13·5 |
| 4 | IF×C57 Bl | 0 | 2 | 0 | 50 | 0 | 0 | 1 | 25 | 1 | 25 | 12·2 |

None of the ovarian tumours induced by MC were macroscopic but they could be identified microscopically as small granulosa-cell tumours. The other ovaries were somewhat atrophied showing changes similar to, but less marked than, those of the mice treated with DMB.

C. *Histological changes induced in mouse ovaries by DMB*

For this report histological material from ovaries of all IF mice receiving DMB treatment was studied. Some mice were killed at intervals of a fortnight after beginning treatment, and other mice which died during the course of the experiments were also used.

The changes noted in the IF mice also occurred in mice of the other strains at about the same rate, except in the A strain, where they seemed to lag about 2 months behind. The changes can most simply be explained under a few headings.

*Normal mouse ovary.*—The bulk of a normal ovary from a young adult mouse is composed of follicles and corpora lutea (Fig. 1). The latter are clearly defined bodies composed of smallish cells with eosinophilic cytoplasm. As these bodies age, they become invaded by fibroblasts from the theca and gradually retrogress. Amongst the corpora lutea are to be found follicles in various stages of development. Some are primary follicles with an ovum surrounded by a single layer of granulosa cells ; others contain granulosa cells which have proliferated into several layers, with some eosinophilic fluid around the ovum in the larger follicles. Still more follicles of various sizes have undergone degeneration or atresia. The

---

EXPLANATION OF PLATES

All figures show sections of ovaries of IF or IF hybrid mice treated fortnightly with DMB, at the same magnification, except Fig. 12.

Fig. 1 to 4 show the early results of DMB treatment.

FIG. 1.—Normal young mouse ovary showing developing follicles. × 26.

FIG. 2.—Two weeks DMB. Many follicles degenerating. × 26.

FIG. 3.—Three and three-quarter months DMB. Only 1 developing follicle present. Outlines of corpora lutea are indistinct. ×26.

FIG. 4.—Four months DMB. No ova remain, lutein tissue is diffuse and patches of pigmented cells have appeared. × 26.

Fig. 5 to 7 show ovaries of mice with early tumours.

FIG. 5.—Four months DMB. Diffuse luteinisation and remains of large degenerated follicles. × 26.

FIG. 6.—Shows an early granulosa-cell tumour in the other ovary of the same mouse as Fig. 5. × 26.

FIG. 7.—Seven and a half months DMB. A small granulosa-cell tumour occupies most of the ovary which is a little enlarged. × 26.

FIG. 8.—Seven and three-quarter months DMB. Atrophied ovary with prominent germinal epithelium. × 26.

Figs. 9 to 11 show various types of cysts.

FIG. 9.—Six months DMB. Papillary cyst typical of Fallopian tube in diffusely luteinised ovary. × 26.

FIG. 10.—Eight and a half months DMB. Haemorrhagic cyst. × 26.

FIG. 11.—Seven and three-quarter months DMB. Part of clear, multiloculate, cystic ovary about 4 mm. diameter. × 26.

FIG. 12.—Eight and a half months DMB. Part of a prominent germinal epithelium enlarged to show " anovular follicles " or " sterile tubules ". × 245.

1

2

3

4

5

6

7

8

9

10

11

12

follicles and corpora lutea form the " cortex " of the ovary and there is a " medulla" composed of blood vessels, fibrous tissue, etc. interspersed with a few atretic follicles. In the normal ovaries of older mice some large vacuolated or pigmented cells may be found.

*Destruction of ova after DMB treatment.*—The first change noticed in the ovaries of mice which have begun DMB treatment was not usually very evident until about one month after the first painting. At this stage many of the ova of follicles of all sizes appeared to be degenerate, resulting in an increase in the numbers of atretic follicles and a gradual decrease in numbers of developing follicles (Fig. 2). In a few of the larger follicles with degenerate ova, luteinisation appeared to be taking place to form " corpora lutea atretica ". After 4 to 5 months of treatment all ova with their surrounding granulosa cells had disappeared, but atretic follicles were still abundant (Fig. 4). The latter gradually decreased in numbers and finally diasppeared after 8 months.

*Disorganisation and disappearance of corpora lutea.*—After about 3 months of treatment with DMB the ovaries were somewhat atrophied, due to loss of developing follicles. Normal corpora lutea were still present, but at about this time they showed a tendency to merge together into large masses of lutein tissue (Fig. 3 and 5) similar to the condition described as " luteomatous " in intrasplenic grafts of guinea-pig ovaries by Iglesias, Mardones and Lipschutz (1953). Later the cells appeared to increase in size and to become less eosinophilic. Eventually larger cells were seen which were often vacuolated or contained pigment (Fig. 4) and identical with the interstitial tissue of other authors. After about 7 months of treatment, normal corpora lutea had disappeared, and also the masses of lutein tissue, leaving clumps of large cells. A few atretic follicles might be seen. Sometimes the ovaries were invaded by lymphocytes and mast cells might be present. On occasion thecal tissue was prominent or blood vessels congested.

*Prominent germinal epithelium.*—After 7 months of treatment, ovaries which did not contain tumours or cysts were considerably atrophied due to the loss of follicles and corpora lutea (Fig. 8). At the same time, and possibly as a result of the atrophy, the germinal epithelial cells became prominent. Instead of being flattened, as in the normal mature ovary, the cells became cuboidal or even columnar in shape and sometimes they were invaginated into the substance of the ovary to form the " sterile tubules" or " anovular follicles " described by other authors, e.g. Brambell, Parkes and Fielding (1927), in irradiated ovaries (Fig. 12).

*Granulosa-cell tumours.*—The first granulosa-cell tumours were found after 4 months from the commencement of DMB treatment. Typical small tumours are shown in Fig. 6 and 7. They were not found in mice surviving for more than 8 months. Presumably, therefore, they must have arisen from some ovarian constituent, which had disappeared before this time. This would seem to exclude the germinal epithelium, which had only begun to proliferate at a time when granulosa-cell tumours had stopped being formed. The most likely origin of the tumours seems to be from the granulosal lining of follicles in which the ova have degenerated as postulated by Traut and Butterworth (1937) and McKay, Hertig and Hickey (1953). Two early tumours have been found in ovaries of mice which still contained one or two large follicles undergoing atresia (Fig. 5 and 6). In mice with tumours in one ovary, the non-tumorous ovary resembled those of the DMB-treated mice without tumours.

*Cysts*.—Cysts of various kinds (Fig. 9 to 11) were found in some ovaries after about 5 months' treatment with DMB. They are sometimes seen in the ovaries of untreated ageing mice.

Table III shows the structures present in the ovaries of mice treated with DMB at various periods after commencement of the treatment.

TABLE III.—*Structures Present in Ovaries of Mice Treated with DMB*

| Months of treatment | Developing follicles (Ova) | Corpora lutea | Disorganised lutein tissue | Large vacuolated or pigmented cells | Atretic follicles | Prominent germinal epithelium | Granulosa-cell tumours | Cysts |
|---|---|---|---|---|---|---|---|---|
| 0 | ++ | ++ | − | ± | + | − | − | − |
| 1 | + | ++ | − | ± | ++ | − | − | − |
| 2 | + | ++ | ± | ± | ++ | − | − | − |
| 3 | + | + | + | ± | ++ | − | − | − |
| 4 | ± | + | ++ | ± | + | − | ± | − |
| 5 | ± | ± | + | + | + | ± | + | ± |
| 6 | − | ± | ± | + | + | ± | + | ± |
| 7 | − | − | ± | ++ | ± | + | ++ | + |
| 8 | − | − | − | ++ | ± | + | ± | + |
| 9 | − | − | − | ++ | − | ++ | − | + |

++ abundant, + present, ± sometimes present, sometimes absent, − absent.

*Vaginal smears*.—In general the vaginal smears of mice treated with DMB showed that they went on cycling for the first four months of treatment, with a tendency towards longer periods of oestrus or of dioestrus, and eventually became fairly constant in one or the other phase. Those mice which finished in a state of fairly constant oestrus usually had a granulosa-cell tumour, occasionally a cyst. Those in constant dioestrus usually had atrophied ovaries, but about one-quarter of the mice with granulosa-cell tumours, and some of those with cysts, were also in constant dioestrus. After a period of dioestrus lasting several weeks, some mice would go into constant oestrus and these were found to have granulosa-cell tumours.

It is seen from Fig. 13 that in general the mice with a history of increasing periods of oestrus had the earlier tumours and it is believed that these tumours began to develop when many viable follicles were present in the ovary. The mice with a history of dioestrus smears, frequently followed by constant oestrus, had the later tumours which probably arose from the last few remaining follicles in an atrophying ovary.

If, after about 5 months' treatment with DMB, a mouse was not in a state of constant dioestrus, an explanation could almost always be found in the form of a granulosa-cell tumour or cyst. On the other hand, mice which were in constant dioestrus after 5 months' treatment were not always free from tumour or cyst.

In the next experiment an attempt was made to find the minimum number of DMB paintings required to induce ovarian tumours. The mice used in this and some of the subsequent experiments were probably not pure IF mice, for about this time some mice with black bellies instead of tan bellies appeared in the strain. It is most likely that they resulted from a cross between IF and C57 Black, so we have designated this strain " CIF ". Another stock of pure IF mice has since been obtained from Dr. G. M. Bonser at Leeds and has been used in all further experiments with the IF strain.

D. *The minimum dose of DMB required to produce ovarian tumours in mice of IF extraction*

Six boxes of virgin female " CIF " mice were given different numbers of fortnightly paintings with the standard DMB solution (from 1 to 6 paintings). They were then kept until they died, or until the condition of the mouse necessitated killing. All ovaries were then examined histologically. Mice surviving less than 4 months are not included in Table IV.

TABLE IV.—*Incidence of Tumours in Mice having Different Numbers of Treatments with DMB*

| Number of DMB treatments | Mice at risk | | Granulosa-cell tumours | | | Breast tumours | Cysts (all +N) | Average survival (months) |
|---|---|---|---|---|---|---|---|---|
| | No. | Strain | +N | N | −N | | | |
| 1 | 6 | " CIF " | 0 | 1 | 1 | 1 | 1 | 16·5 |
| 2 | 5 | " CIF " | 2 | 2 | 0 | 1 | 0 | 15·1 |
| 3 | 3 | " CIF " | 0 | 0 | 0 | 0 | 0 | 4·0 |
| 4 | 2 | " CIF " | 0 | 0 | 1 | 0 | 0 | 4·3 |
| 5 | 6 | " CIF " | 1 | 4 | 0 | 4 | 0 | 10·0 |
| 6 | 6 | " CIF " | 0 | 0 | 0 | 1 | 1 | 6·0 |

The survival of the mice which had 3 and 4 paintings respectively was not good, because of an infective paralysis which usually ended in death. Some of those having 6 paintings also died early from enteritis. Nearly all of the tumours were small, but detectable macroscopically by inequality in size of the ovaries. One very large tumour occurred amongst the mice having 5 paintings and 2 amongst those having 2 paintings.

Although only one painting with DMB is required to induce granulosa-cell tumours of the ovary in a proportion of mice of IF extraction, it seemed that the fewer the paintings the more variable the pathology of the ovaries. Two of the mice which had one painting and two having 2 paintings of DMB had areas of " theca-fibroma " in their ovaries, and in one of the latter group this was not associated with a granulosa-cell tumour.

Ovarian tumours have been produced experimentally in various species of animals by other authors. There are two methods of doing so. Biskind and Biskind (1944) induced them by grafting ovaries into the spleens of castrated rats. It has generally been accepted that such tumours result from an increase of gonadotrophins from the pituitary which enhance the growth of granulosa cells. The increase is due to the destruction in the liver of oestrogens produced by the transplanted ovaries. Mühlbock (1951*b*) has shown by parabiosis experiments that methods of destroying the circulating oestyogen more efficiently will reduce the induction time for ovarian tmours. No tumours are produced in the intrasplenic grafts if one ovary of the animal is left intact (Biskind and Biskind, 1948) or in castrates receiving oestradiol or testosterone, but not in castrates receiving progesterone, after grafting (Li and Gardner, 1949).

Ovarian tumours may also be induced by X-radiation (Furth and Butterworth, 1936). Intact endocrine function has been found to inhibit development of tumours in irradiated ovarian grafts (Kaplan, 1950), but Gardner (1950) found that although administration of oestradiol benzoate prevented the induction of ovarian tuours by X-rays, testosterone propionate did not. Chang and Eck (1952) found that the dosage of testosterone propionate used by Gardner was sufficient to prevent hyperactivity of the pituitary and concluded that some factors other than overproduction of gonadotrophins must be responsible for the occurrence of ovarian tumours in X-rayed animals.

We desired to know whether the administration of sex hormones would prevent the induction of ovarian tumours in IF mice by DMB, so the following experiments were performed.

E. *The influence of sex hormone administration concurrently with DMB treatment*

(i) *DMB alone.*—Seven young adult female " CIF " virgin mice were given DMB to see whether these mice were susceptible to ovarian tumour production.

The results are given in Table V. The mice survived an average of 7·3 months of treatment (range 6 to 9 months). Two of these mice developed large granulosa-cell tumours of the ovary after 6·8 months. One of them also had a breast tumour. Two of the mice without ovarian tumours had breast tumours. Sections of the non-tumorous ovaries showed large pigmented cells, but no follicles or corpora lutea.

TABLE V.—*Influence of Sex Hormone Administration on the Incidence of Tumours induced with DMB*

| Treatment (fortnightly) | Mice at risk | | Number with granulosa-cell tumours | | | | Number with ovarian cysts only | | | | Number with breast tumours | | Survival (months) |
|---|---|---|---|---|---|---|---|---|---|---|---|---|---|
| | No. | Strain | +N | N | −N | % | +N | N | −N | % | | % | |
| DMB | 7 | " CIF " | 2 | 0 | 0 | 28 | 0 | 0 | 0 | 0 | 3 | 43 | 7·3 |
| DMB +Oestrogen | 7 | " CIF " | 1 | 3 | 0 | 57 | 0 | 1 | 0 | 14 | 2 | 28 | 7·7 |
| Oestrogen | 9 | " CIF " | 0 | 0 | 0 | 0 | 0 | 1 | 0 | 11 | 0 | 0 | 11·5 |
| DMB | 43 | IF | 11 | 6 | 9 | 60 | 0 | 1 | 0 | 2 | 33 | 77 | 6·7 |
| DMB +progesterone | 20 | IF | 1 | 3 | 2 | 30 | 0 | 0 | 0 | 0 | 9 | 45 | 4·7 |
| DMB +testosterone | 13 | IF | 1 | 1 | 0 | 15 | 0 | 1 | 0 | 8 | 2 | 15 | 4·7 |

(ii) *Oestrogen alone.*—Nine virgin female " CIF " mice were treated with 0·2 per cent stilboestrol dipropionate in olive oil for 11·5 months. None developed granulosa-cell tumours, but one small ovarian cyst was fund. No breast tumours were encountered in these mice. Histologically their ovaries were seen to contain some follicles, but few or no corpora lutea.

(iii) *Oestrogen and DMB.*—Seven young adult virgin " CIF " mice were painted in the usual way with olive oil containing 0·5 per cent DMB and 0·02 per cent stilboestrol dipropionate. Treatment was continued until the mice died or were killed because of their poor condition.

As will be seen from Table V the mice survived an average of 7·7 months (range 5·5 to 10·5 months) of treatment. Two of the 7 mice died after 5·5 months and had no ovarian tumours, but 1 had a breast tumour. Four mice dying after 6·5, 6·8, 9·5 and 9·5 months respectively had granulosa-cell tumours in one ovary. The earliest of these tumours measured approximately 2·5 cm. in diameter; the other 3 were only detectable by slight inequality of the ovaries but they were confirmed histologically. One of these mice also had a breast tumour. The seventh mouse dying after 10·5 months had a small cyst in one ovary. Thus 4 out of 7 mice at risk developed granulosa-cell tumours of the ovary after a mean time of

8 months' treatment with DMB and stilboestrol dipropionate simultaneously. Non-tumorous ovaries contained pigmented cells and were devoid of developing follicles and corpora lutea.

It appears from the above results that oestrogen administration concurrently with DMB treatment does not inhibit the production of granulosa-cell tumours of the ovary.

(iv) *Progesterone and DMB.*—In this experiment 20 pure line IF mice were painted fortnightly with olive oil containing 0·5 per cent DMB and 2 per cent progesterone.

The survival of these mice was not good. Many of them developed lymphomata and others had extensive liver necrosis. The mean survival time was 4·7 months (range 4 to 5·5 months). Six of these mice developed granulosa-cell tumours. One was large, three detected by inequality in size of the ovaries, and two were only detected microscopically. Nine mice developed breast tumours. In the non-tumorous ovaries only corpora lutea, atretic follicles and some large cells were seen.

It appears then that concurrent administration of progesterone does not prevent the induction of ovarian tumours by DMB.

(v) *Androgen and DMB.*—Thirteen pure IF strain virgin female mice were used in this experiment. They were painted fortnightly with olive oil containing 0·5 per cent DMB and 1 per cent testosterone propionate.

These mice were also poor survivors, the mean survival time being 4·7 months (range 3·5 to 7·5 months). About half of them developed paralysis of the hind legs, from which some recovered, but were found to have enlarged axillary or abdominal lymph nodes at autopsy. In spite of the short survival, 3 mice developed granulosa-cell tumours. One, in a mouse which died after 4·5 months' treatment, was very large, but the other 2 were only detected microscopically. One of the latter was in a mouse which had received only 3·5 months' treatment and was the earliest tumour ever seen in DMB-treated mice. The non-tumorous ovaries showed diffuse luteinisation with a few atretic follicles. One mouse had a cystic ovary. Two mice developed breast tumours, one of these being the mouse with the earliest ovarian tumour.

Concurrent administration of androgen does not prevent the induction of ovarian tumours by DMB.

It has previously been shown that breeding and lactation exerts a protective effect against the production of breast tumours in IF mice treated with an oily solution of MC as in the above experiments (Marchant, 1955). In view of the production of profound changes in the ovaries of mice by DMB it was decided to determine what effect DMB treatment would have on breeding mice.

F. *The effect of DMB on breeding mice*

(i) *Fortnightly treatment of breeding IF mice with DMB.*—Fifteen female IF mice between 2 and 3 months of age were mated in individual boxes and allowed to breed indefinitely. After the first litter had been suckled, DMB paintings were commenced. Further litters were noted when they occurred.

One of the 15 treated mice did not produce another litter, 4 produced 1 litter each, 4 produced 2 litters and 6 produced 3 further litters. No litters were born more than 66 days after the first DMB painting. All the mice had ceased to breed by the age of 5 months, whereas normal mice of this strain can be expected to continue breeding until at least twice this age. No abnormalities were noted amongst the offspring, but they were not kept after weaning age.

The ovaries of 9 of the 15 mice, which survived from 4·5 to 7 months (mean 6 months) of the treatment with DMB, were studied histologically. Small granulosa cell tumours were detected in only 2 cases. Other ovaries were very atrophied and did not contain ova or follicles other than atretic ones. The general picture was similar to that of ovaries of virgin mice treated for a similar period. Breast tumours occurred in 2 of the 9 surviving mice treated with DMB, both of which had 3 litters after DMB painting, and these 2 mice were the ones in which the ovarian tumours were detected.

Table VI compares these mice with virgin mice treated with DMB and with breeders and virgins treated with MC in earlier experiments (Marchant, 1955). A $\chi^2$ test on these data showed that breeding did not lower the incidence of granulosa-cell tumours in IF mice treated with MC, and in those treated with DMB it was not lowered significantly ($P > 0{\cdot}1$). On the other hand, breeding did lower the incidence of breast tumours significantly in mice treated with either carcinogen ($P < 0{\cdot}01$ in both cases).

TABLE VI.—*Incidence of Tumours in Virgin and Breeding IF Mice Treated with MC or DMB*

| Treatment | Mice at risk | | Mean number litters after 1st painting (range) | Number with granulosa-cell tumours of ovary | | | | Number with ovarian cysts only | | | | Number with breast tumours | | Survival (months) |
|---|---|---|---|---|---|---|---|---|---|---|---|---|---|---|
| | No. | Strain | | +N | N | −N | % | +N | N | −N | % | | % | |
| MC | 19 | Virgin IF | 0 | 0 | 0 | 1 | 5 | 0 | 3 | 2 | 20 | 14 | 74 | 10 |
| MC | 11 | Breeding IF | 3 (1–5) | 0 | 0 | 0 | 0 | 0 | 0 | 0 | 0 | 0 | 0 | 8 |
| DMB | 43 | Virgin IF | 0 | 11 | 6 | 9 | 60 | 0 | 1 | 0 | 2 | 33 | 77 | 6·7 |
| DMB | 9 | Breeding IF | 2 (0–3) | 0 | 2 | 0 | 20 | 0 | 0 | 0 | 0 | 2 | 20 | 6 |

Both hydrocarbons have a marked carcinogenic effect on the breast of virgin IF, but DMB produces significantly more granulosa-cell tumours of the ovary than MC ($P < 0{\cdot}01$).

(ii) *A single treatment of breeding albino mice with DMB.*—In this experiment 13 outbred albino mice were mated 2 or 3 females to 1 male in a box. All the females received a single painting of DMB either before or after producing their first litter. The males were not removed.

Since it was not always certain which mouse produced which litter, only the totals are given. These 13 mice produced 27 litters in all after the DMB treatment, a mean of 2·1 litters each. The latest litter was produced 6·5 months after the painting. Mice of this stock often produce as many as 10 litters with a large average number in each litter, so fertility was certainly reduced. At the time of writing 6 of these mice have died with breast tumours, but none had ovarian tumours.

(iii) *Fortnightly treatment of breeding albino mice with* 2 : 7-1 : 2 *DMB* (*non-carcinogenic*).—In this experiment 16 breeding albino mice were treated once a fortnight with a 0·5 per cent solution of the non-carcinogenic 2 : 7-dimethyl-1 : 2-

benzanthracene to see whether this substance had any effect on fertility. The mice were mated with 2 females to 1 male.

After the first painting a total of 77 litters was produced by the 16 mice with a mean of 4·8 litters each. By this time it was obvious that breeding had not been reduced and the males were removed from the females 5·5 months after the first painting. The females are still under observation.

TABLE VII.—*Effect of DMB on Fertility of Mice*

| Treatment | Mice used No. | Mice used Strain | Mean number litters after painting | Time of last litter (from 1st painting) (months) |
|---|---|---|---|---|
| DMB fortnightly | 15 | IF | 2 | $2\frac{1}{4}$ |
| DMB once | 13 | Outbred albino | 2·1 | $6\frac{1}{2}$ |
| 2·7-DM-1·2-B fortnightly | 16 | Outbred albino | 4·8 (male removed) | $6\frac{1}{4}$ (male removed) |

Table VII summarises the results of these experiments with DMB on fertility.

It is seen that, whereas a single painting of 9 : 10-dimethyl-1 : 2-benzanthracene (DMB) was capable of reducing the fertility of breeding albino stock females, continuous fortnightly paintings of 2 : 7-dimethyl-1 : 2-benzanthracene had no apparent effect. IF mice treated fortnightly with DMB no longer produced litters after $2\frac{1}{4}$ months from the first treatment.

### DISCUSSION

### *The effect of DMB treatment on mouse ovaries*

It is evident from Experiment C above that the histological changes which took place in the ovaries of mice treated with DMB were very similar to those occurring after X-radiation, as described by Traut and Butterworth (1937). After both, ova degenerated, then granulosa cells and finally lutein cells much more slowly. Then the germinal epithelium hypertrophied. Granulosa-cell tumours probably arose fairly early, apparently from surviving granulosa cells in a partially degenerated follicle. Unlike the X-rayed ovaries, however, those subjected to the influence of DMB did not show any tendency to produce luteomas.

Although the incidence of tumours after DMB varied so greatly in the different strains of mice used (Experiment A), the degenerative changes occurred in all strains and were very similar. This would appear to indicate that tumour production by DMB is not mediated through the hormonal imbalance which must eventually result from these degenerative effects.

There appears to be some variability in incidence of ovarian tumours in two different strains of mice subjected to X-rays (Mühlbock 1951*a*), granulosa-cell tumours being obtained in the Little dilute-brown strain, but not in the C57 Black.

The primary action of DMB on mouse ovaries seems to be oocyte destruction, and this is also the primary action of X-rays on the ovary. It has also been found (Little *et al.*, 1951) that the other method of inducing ovarian tumours, by intra-splenic grafting, appears to affect permanently the ability of the ovary to produce viable ova.

*Minimum dose of DMB required to induce ovarian tumours*

Experiment D shows that only one treatment with DMB was sufficient to obtain some tumours. If one discounts the mice which died from infectious disease, it would seem that the yield of tumours after small numbers of paintings was good and the mice survived longer. It is possible, however, that the induction time was longer after fewer paintings.

*Secretory activity of tumour tissue*

Many of the granulosa-cell tumours induced by DMB apparently secreted oestrogen and gave oestrus vaginal smears. To some extent the oestrogenic activity of the tumours was probably related to their size, for in some cases the oestrus smears were preceded by a long phase of dioestrus during which the tumour was presumably present and growing (Fig. 13). But a small number of large

Fig. 13.—Relationship between oestrus history, duration of DMB treatment and size of tumorous ovaries.

Each rectangle represents one dead mouse with an ovarian tumour.

tumours were not associated with oestrus vaginal smears. These non-oestrogenic tumours were not histologically different from oestrogenic ones.

The fact that granulosa-cell tumours are usually, but not always, associated with oestrus vaginal smears has been noted by other authors (Biskind and Biskind, 1949) in tumours arising from intrasplenic grafts.

*The effect of simultaneous sex hormone administration on tumour induction by DMB*

It is evident from Experiment E that the amounts of stilboestrol dipropionate, progesterone or testosterone propionate administered along with DMB did not prevent the induction of ovarian tumours by the carcinogen in mice of IF stock. This is different from the experience of authors inducing ovarian tumours by X-rays or intrasplenic grafting, for in both cases oestrogens (and, in the case of intrasplenic grafting, testosterone also) prevented their appearance (Li and Gardner

1949 ; Gardner, 1950). In Experiment E the amount of stilboestrol administered was sufficient to suppress luteinisation in normal mice, but there was no reduction in ovarian tumour incidence in mice given the oestrogen together with DMB. It seems that DMB can induce ovarian tumours by some action apart from its interference with the sex-hormone balance of the animals.

*Comparison of the carcinogenic effects of DMB and MC*

Experiment B indicates that the fortnightly treatment of virgin mice of IF stock with MC does affect the ovaries, as well as producing the high incidence of breast tumours previously reported by Orr (1946). However, significantly fewer lesions were produced after MC treatment which were identical with the small granulosa-cell tumours seen after DMB treatment. No large ovarian tumours were formed and the atrophic changes did not proceed so rapidly as with DMB. It seems then that DMB is more powerful in its carcinogenic effect on the ovaries of the IF mice than MC, while they are to all intents and purposes equally effective on the breasts.

Experiment F showed that breeding significantly lowered the incidence of breast tumours in IF mice treated with either carcinogen. Although breeding did not significantly lower the incidence of ovarian tumours after DMB treatment it is interesting that, in the breeding mice only those which had developed ovarian tumours also developed breast tumours.

*The influence of DMB on fertility*

Although DMB seemed to be really effective in inducing ovarian tumours only in mice of IF origin, it did have a marked sterilising effect on the ovaries of all the mouse strains used in these experiments, producing degeneration of ova and follicles, followed by disappearance of corpora lutea. It did not affect rat ovaries, however, in a series of experiments carried out in this department.

The Experiment, F in which DMB was given to breeding IF mice, showed similar results. After 66 days from the commencement of treatment, no further litters were produced. There is no reason to believe that similar sterilising effects would not be achieved in other strains of mice with DMB as a result of its destructive effect on ova.

Experiment F also shows that the non-carcinogenic 2 : 7-dimethyl-1 : 2-benzanthracene, which differs from the carcinogenic 9 : 10-dimethyl-1 : 2-benzanthracene (DMB) only in the positions of the two methyl groups, has no deleterious effect on the breasts or ovaries of mice.

### SUMMARY

1. A study has been made of the induction of granulosa-cell tumours of the ovary in mice of various strains by fortnightly painting with a solution of 0·5 per cent 9 : 10-dimethyl-1 : 2-benzanthracene (DMB) in olive oil.

2. Whereas an incidence of 40 to 70 per cent of granulosa-cell tumours is found in mice of the IF strain and $F_1$ hybrids of this strain, the following incidences were obtained in other strains : C57 Bl—11 per cent, outbred albino—18 per cent, and none in A or C3H.

3. No normal ovaries were found in any mice which received DMB treatment. After 4 months' treatment all ova had degenerated and developing follicles disappeared. Corpora lutea disappeared about two months later, leaving an atrophied ovary with a prominent germinal epithelium in non-tumorous ovaries. This applied to all the strains of mouse except the A strain in which the changes lagged about two months behind.

4. Ovaries of IF mice similarly treated with 0·5 per cent 20-methylcholanthrene in olive oil showed less-marked but similar changes, and a small number of ovarian tumours was found on histological examination.

5. Only one treatment with DMB was necessary to induce granulosa-cell tumours in mice of IF origin.

6. The administration of the following sex hormones simultaneously with the 0·5 per cent DMB did not prevent the induction of granulosa-cell tumours in mice of the IF strain and related hybrids : 0·02 per cent stilboestrol dipropionate, 2 per cent progesterone, and 1 per cent testosterone propionate—all administered in the olive oil containing the DMB.

7. Breeding significantly lowered the incidence of breast tumours in IF mice treated with DMB, tumours only occurring in those mice which also had ovarian tumours.

8. The fortnightly DMB treatment of breeding IF mice rendered them sterile in a little over 2 months. A single treatment with DMB markedly reduced the fertility of breeding albino stock mice, but fortnightly treatment of similar mice with 0·5 per cent 2 : 7-dimethyl-1 : 2-benzanthracene had no apparent effect on breeding.

This work was supported by the Birmingham Branch of the British Empire Cancer Campaign.

### REFERENCES

Biskind, G. R. and Biskind, M. S.—(1944) *Proc. Soc. exp. Biol. N.Y.*, **55**, 176.—(1948) *Science*, **108**, 137.—(1949) *Amer. J. clin. Path.*, **19**, 501.
Brambell, F. W. R., Parkes, A. S. and Fielding, U.—(1927) *Proc. Roy. Soc.* B., **101**, 29.
Chang, C. H. and Eck, G. V. van.—(1952) *Cancer Res.*, **12**, 254.
Furth, J. and Butterworth, J. S.—(1936) *Amer. J. Cancer*, **28**, 66.
Gardner, W. U.—(1950) *Proc. Soc. exp. Biol. N.Y.*, **75**, 434.
Howell, J. S., Marchant, J. and Orr, J. W.—(1954) *Brit. J. Cancer*, **8**, 635.
Iglesias, R., Mardones, E. and Lipschutz, A.—(1953) *Ibid.*, **7**, 214.
Kaplan, H. S.—(1950) *J. nat. Cancer Inst.*, **11**, 125.
Li, M. H. and Gardner, W. U.—(1949) *Cancer Res.*, **9**, 35.
Little, C. C., Hummel, K. P., Eddy, M. and Rupple, B.—(1951) *Proc. nat. Acad. Sci., Wash.*, **37**, 666.
McKay, D. G., Hertig, A. T. and Hickey, W. F.—(1953) *Obstet. Gynec.*, **1**, 125.
Marchant, J.—(1955) *J. Path. Bact.*, **70**, 415.
Mühlbock, O.—(1951*a*) *Ned. Tijdschr. Geneesk.*, **95**, 915.—(1951*b*) *Ibid.*, **95**, 3672.
Orr, J. W.—(1946) *J. Path. Bact.*, **58**, 589.
Traut, H. F. and Butterworth, J. S.—(1937) *Amer. J. Obstet. Gynec.*, **34**, 987.